# Tehran Dust Storm Early Warning System: Corrective Measures

**DOI:** 10.1371/currents.dis.14f3c645eb2e2003a44c6efd22c23f5e

**Published:** 2015-02-24

**Authors:** Mohammad Javad Moradian, Behnaz Rastegarfar, Mohammad reza Rastegar, Ali Ardalan

**Affiliations:** Department of Disaster Public Health, School of Public Health, Tehran University of Medical Sciences, Tehran, Iran; Department of Disaster and Emergency Health, I.R.Iran’s National Institute of Health Research, Tehran University of Medical Sciences, Tehran, Iran; Education Development Center, Shiraz University of Medical Sciences, Shiraz, Iran; Department of Disaster & Emergency Health, Iran's National Institute of Health Research; Department of Disaster Public Health, School of Public Health, Tehran University of Medical Sciences, Tehran, Iran; Harvard Humanitarian Initiative, Harvard University, Cambridge, Massachusetts, USA

**Keywords:** dust storm, early warning system, Tehran

## Abstract

On June 2, 2014 a sandstorm hit Tehran, the capital city of Iran which killed 5 and injured 44 people. The early warning system did not operate properly and the alarm was not transferred to at risk population and the related organizations in time and in a right manner. Additionally, people who were exposed to the winds didn't know the appropriate safety measures. Focusing much more on establishing EWS to alert the risk prone population timely and public education for taking safety measures when exposed to the disastrous situation is recommended.

## Introduction

Dust storms, which is wind carrying suspended loose sand from a dry surface, are common in arid and semi-arid areas like Iran but rare in Tehran, and planning is needed in order to be prepared for such disasters 1.On June 2, 2014 a sandstorm hit Tehran, the capital city of Iran at 17:15 which was a rush hour in the afternoon when people were heading home. This 110 kilometer (70-mile) per hour wind, which lasted more than 15 minutes, disrupted many functions of the city 2 ^,^ 3. In 1997 in eastern Mediterranean and also 2004 in China, similar incidents with sudden change in the air mass occurred. After the eastern Mediterranean dust storm they found that employing a synoptic network would make it feasible to forecast dust events up to three days before its progression to a significant one 4 ^,^ 5. Xueqin Liu et al, in their study found that in incidents such as extremely severe dust storms which occurred in the Alashan League in the spring of 1993, losses reached an unacceptable level. They declared that economic and other losses might be prevented by planning on disaster prevention, monitoring, and early warning system 6.

Establishing early warning system (EWS) is one of the main parts of disaster response planning which includes four elements: 1- hazard identification, 2-hazard monitoring, 3-sending alert to the risk prone population, and 4-building response capacity 7. This report focuses mostly on sending alerts and enhancing response capacity to present the most important lessons learned at Tehran dust storm in 2014.

## Method

Data for this report were collected through Tehran emergency operations center reports and documents, interview with the manager of Tehran Emergency Management Center and from news websites. It was backed up with literature review through "PubMed" with the following key word: "dust storm” and “early warning”.

## Results

This incident had a death toll of five (two persons because of falling from buildings, two persons due to falling objects and one is unknown) and 44 injured, who mostly were surprised by the powerful winds. Other impacts were knocked down trees, floating debris, car crashes due to low visibility, temporary power outages, affected Internet and telephone services, delayed flights, and damaged buildings 3. Local news agencies notified that the head of meteorological organization had warned the disaster management organization of Tehran municipality a few minutes before the storm reached the capital city. News agencies also claimed that shortly before the incident, forecasters on the state television initially warned citizens of Tehran to stay indoors, while outdoor people were much at risk. In this incident, the alarm was not transferred to at risk populations and the related organizations in time and in the right manner. Additionally, no safety measures regarding this type of incident had been taught to the people to be taken after the alert had been received.

## Discussion

Although the possibility of prediction of such incidents is challenging and needs high technological infrastructure, warning other related organizations after the formation of storm might have been possible 8.

Despite being a rare incident in Tehran, dust storms are common in Iran 9. Therefore, the lesson learned is that for such rare high impact incidents which have short warning time, response planning should focus much more on establishing EWS to give timely alert to the risk prone population. For example, to cover all target population including those staying outdoors, preplanned instruments such as sirens are needed along with routine media broadcasting.

Finally, public education for taking safety measures after receiving the alert or when exposed to the disastrous situation, is recommended towards developing a community-based disaster response.

## Competing Interests

The authors have declared that no competing interests exist.

## Correspondence

Ali Ardalan MD, PhD. Tehran University of Medical Sciences. Harvard Humanitarian Initiative. Email: ardalan@hsph.harvard.edu
